# Tumor-stroma metabolic relationship based on lactate shuttle can sustain prostate cancer progression

**DOI:** 10.1186/1471-2407-14-154

**Published:** 2014-03-05

**Authors:** Patrizia Sanità, Mattia Capulli, Anna Teti, Giuseppe Paradiso Galatioto, Carlo Vicentini, Paola Chiarugi, Mauro Bologna, Adriano Angelucci

**Affiliations:** 1 Department of Biotechnological and Applied Clinical Sciences, University of L’Aquila, Via Vetoio, Coppito 2, 67100 L’Aquila, Italy; 2 Department of Life, Health and Environmental Sciences, University of L’Aquila, 67100 L’Aquila, Italy; 3 Department of Biochemical Sciences, Tuscany Tumor Institute and “Center for Research, Transfer and High Education DenoTHE”, University of Florence, 50134 Florence Italy

**Keywords:** Aerobic glycolysis, Monocarboxylate transporters, Cancer associated fibroblasts, Warburg effect, Tumor stroma

## Abstract

**Background:**

Cancer cell adopts peculiar metabolic strategies aimed to sustain the continuous proliferation in an environment characterized by relevant fluctuations in oxygen and nutrient levels. Monocarboxylate transporters MCT1 and MCT4 can drive such adaptation permitting the transport across plasma membrane of different monocarboxylic acids involved in energy metabolism.

**Methods:**

Role of MCTs in tumor-stroma metabolic relationship was investigated in vitro and in vivo using transformed prostate epithelial cells, carcinoma cell lines and normal fibroblasts. Moreover prostate tissues from carcinoma and benign hypertrophy cases were analyzed for individuating clinical-pathological implications of MCT1 and MCT4 expression.

**Results:**

Transformed prostate epithelial (TPE) and prostate cancer (PCa) cells express both MCT1 and MCT4 and demonstrated variable dependence on aerobic glycolysis for maintaining their proliferative rate. In glucose-restriction the presence of L-lactate determined, after 24 h of treatment, in PCa cells the up-regulation of MCT1 and of cytochrome c oxidase subunit I (COX1), and reduced the activation of AMP-activated protein kinase respect to untreated cells. The blockade of MCT1 function, performed by si RNA silencing, determined an appreciable antiproliferative effect when L-lactate was utilized as energetic fuel. Accordingly L-lactate released by high glycolytic human diploid fibroblasts WI-38 sustained survival and growth of TPE and PCa cells in low glucose culture medium. In parallel, the treatment with conditioned medium from PCa cells was sufficient to induce glycolytic metabolism in WI-38 cells, with upregulation of HIF-1a and MCT4. Co-injection of PCa cells with high glycolytic WI-38 fibroblasts determined an impressive increase in tumor growth rate in a xenograft model that was abrogated by MCT1 silencing in PCa cells. The possible interplay based on L-lactate shuttle between tumor and stroma was confirmed also in human PCa tissue where we observed a positive correlation between stromal MCT4 and tumor MCT1 expression.

**Conclusions:**

Our data demonstrated that PCa progression may benefit of MCT1 expression in tumor cells and of MCT4 in tumor-associated stromal cells. Therefore, MCTs may result promising therapeutic targets in different phases of neoplastic transformation according to a strategy aimed to contrast the energy metabolic adaptation of PCa cells to stressful environments.

## Background

Tumors have long been known to exhibit altered metabolic profiles and increased energy requirements. In fact, the high rate in cell proliferation associated with cancer growth requires a continuous production of ATP and cofactors, consuming glucose in excess. The exemplificative manifestation of such metabolic reprogramming is the formation of lactic acid even in presence of oxygen, a phenomenon referred as “aerobic glycolysis” or the “Warburg effect” [1]. Glycolysis has been also observed in cancer cells without defects in oxidative metabolism, suggesting that it may provide effective advantages for proliferating cells in both bioenergetics and biosynthesis [2]. Growth factors, hypoxia and oncogenes stimulate glycolysis and L-lactate production and are sufficient to induce the Warburg effect in either non-transformed cells or cancer cells [3]. In addition, cancer cell metabolism demonstrates a high adaptability to changing environmental conditions, permitting the continuous cancer growth in fluctuating oxygen tension and glucose concentration. These metabolic changes are thought to be important hallmarks of cancer, and when occurring early during neoplastic transformation, may provide useful biomarkers and targets for intervention [4].

Monocarboxylate transporters (MCTs) are critical for supporting the radical alterations seen in cancer cell metabolism. MCT1 and MCT4, the best characterized members of MCT family, are proton-linked isoforms, which mediate in humans the transport of a range of monocarboxylic acids, including L-lactate, pyruvate, butyrate and ketones, across the plasma membrane of several cell types [5]. The differences in histologic distribution and kinetic activities are at the basis of their specific physiologic roles. This aspect is well represented in skeletal muscles, where L-lactate is exported prevalently by MCT4-expressing glycolytic fibers and it is imported and utilized by MCT1-expressing oxidative muscle fibers [6].

MCT1 was reported to have an ubiquitous tissue distribution, and its expression is stimulated in response to increased metabolic request or to the presence of substrates [7,8]. MCT4 is expressed prevalently in those glycolytic cells that export large amounts of lactic acid and it is transcriptionally upregulated by hypoxia-induced transcription factor, HIF-1. However, recent studies on the role of L-lactate in normal metabolism have elucidated that hypoxia is not a necessary requirement for glycolysis and MCT4 expression. In fact, independently from hypoxia, within tissues such as brain and ovary, some cells become active L-lactate producers, while other cells utilize L-lactate as mobile fuel for aerobic metabolism [9,10]. Accumulation of L-lactate has been frequently associated also with cancer progression and it was correlated to increased metastasis and poor disease-free and overall survival [11]. In parallel, upregulation of MCT1 and MCT4 has been reported in several cancers, including colon, breast and lung cancer [12], and it was associated with the possibility to exchange L-lactate between different cancer cells or between cancer and stromal associated cells, a mechanism called “reverse Warburg effect” [13,14].

Prostate cancer (PCa) is usually a slow-growing malignancy: hence the problem emerges of determining which tumors demonstrate an advantage in energy metabolism. This fact may have important consequences for therapeutic management of PCa, preventing unnecessary treatment in patients for whom the disease is not life threatening. Neoplastic transformation in prostate cells coincides with restoration of full functionality in Krebs cycle, and consequent increased generation of ATP from glucose oxidation and low citrate levels compared to normal prostate [15]. Moreover, PCa is characterized by high levels of L-lactate [16] and this has been linked to the presence of hypoxic regions [17]. The hypoxia can induce a selective pressure toward the glycolytic metabolism and L-lactate production. However the molecular mechanisms and the clinical impact of the metabolic changes observed during prostate neoplastic transformation are largely unknown.

In our study we aimed to elucidate the distribution and the functional role, with particular regard to L-lactate utilization, of MCT1 and MCT4 in PCa. For this reason we investigated in vitro and in vivo the role of MCTs in PCa cell and transformed prostate epithelial cells, and verified the potential role of MCT1 as target in PCa therapy. In addition we analyzed by immunostaining the MCTs expression in PCa and benign prostate hypertrophy (BPH) tissue specimens.

## Methods

### Patients

A total of 140 patients diagnosed for PCa (N = 80) and benign prostate hypertrophy (BPH) (N = 60) and requiring surgical treatment, were enrolled in our Urology Clinic, Department of Medicine, the University of L’Aquila. The research has been carried out in accordance with the Declaration of Helsinki and approved by the Internal Ethical Board of University of L’Aquila. Consent was obtained from all patients after full explanation of the purpose of the study. The adhesion to the study did not implicate any modification in the routine clinical management of the patients. Inclusion criteria were: patients affected by PCa or BPH in the age between 50 and 80 years and a body mass index (BMI) between 25 and 30 (the most frequent range). The diagnosis of BPH was confirmed by the histopathological analysis of the tissue obtained after transvescical retropubic adenomectomy (TV-adenomectomy) or transurethral resection of prostate (TURP). PCa was diagnosed by routine biopsy procedure and the presence and the extension of the tumor was evaluated on the entire gland after the prostatectomy. A detailed clinical history including smoking habit, alcohol abuse, pharmacological therapies as well as comorbidities was obtained for each patient enrolled. Systemic blood samples were drawn from overnight-fasting patients and used to measure PSA, testosterone and fasting insulin through routine analysis performed by our clinical laboratory.

### Animals and experimental in vivo model

Male CD1 nude mice (Charles River, Milan, Italy) were maintained under the guidelines established by our Institution (University of L’Aquila, Medical School and Science and Technology School Board Regulations, complying with the Italian government regulation n.116, January 27 1992 for the use of laboratory animals) and approved by Internal Ethical Board of University of L’Aquila. Before any invasive manipulation, mice were anesthesized with a mixture of ketamine (25 mg/ml)/xylazine (5 mg/ml). Xenografts were obtained by injecting s.c. 1 × 10^6^ tumor cells in 500 μl of phosphate buffer saline. In groups receiving both tumor cells and fibroblasts, a ratio of 1:3 (tumor cells/ fibroblasts) was used. Metformin (Sigma, St. Louis, MI, USA) was dissolved in cell culture medium and was administered at a dose of 50 mg/ kg every other day by intraperitoneal injection, with the appropriate diluent made up to a total volume of 200 μl. Tumor growth was monitored daily by measuring the average tumor diameter (two perpendicular axes of the tumor were measured by a caliper). The volume of the tumor was expressed in mm^3^ according to the formula: volume = (width)^2^ × length/2.

### Immunohistochemistry

Tissue samples were fixed in 4% formaldehyde in 0.1 M phosphate buffer, pH 7.2 and embedded in paraffin. Slide-mounted tissue sections (4-μm thick) were deparaffinized in xylene and serially hydrated in 100%, 95%, and 80% ethanol. Endogenous peroxidases were quenced in 3% H_2_O_2_ in phosphate-buffered saline (PBS) for 1 h and then slides were incubated with an anti-human primary antibody (10 μg/ml) for 1 h and then with peroxidase-conjugated secondary antibody for 30 min at room temperature (RT). Sections were washed three times in PBS and antibody binding was revealed using the Sigma fast 3,30-diaminobenzidine tablet set (Sigma). Counterstaining was performed using haematoxylin solution. Anti-human MCT1 (H-70), anti-human MCT4 (H-90) and secondary antibodies were purchased from Santa Cruz Biotechnology (Santa Cruz, CA, USA). The expression of MCTs was quantified using Remmele scoring system [18]. The score was calculated by multiplying the number reflecting the dominant stain intensity (0, no detectable stain; 1, weak stain; 2, moderate stain; or 3, strong stain) by the number reflecting the percentage of these positive tumor cells (0, no positive cells; 1, <10%; 2, 10-50%; 3, 51-80%; or 4, >80%). The 12-point scale was categorized in three expression groups: 0 = no expression; 1–5 = weak expression; 6–12 = high expression.

### Cell culture

LNCaP, human prostate cancer cell line isolated from metastatic lymph node, was cultured in RPMI 1640 supplemented with 10% v/v fetal bovine serum (FBS), 10 mM HEPES, 1 mM sodium pyruvate, 2 mM glutamine, 100 lU/ml penicillin, and 100 μg/ml streptomycin (Sigma). PC3, human prostate cancer cell line isolated from bone metastasis, was cultured in Coon’s modified Ham’s medium supplemented with 10% FBS, 2 mM glutamine and penicillin-streptomycin. RWPE-1 and WPE1-NB26, human prostate epithelial cells transformed by human papillomavirus-18, were cultured in keratinocyte serum-free medium (K-SFM) with 5 ng/ml EGF and 0.05 mg/ml bovine pituitary extract, BPE (Gibco, Life Technologies Corporation, Grand Island, NY, USA). Human embryonic fibroblasts WI-38 were cultured in Dulbecco’s Modified Eagle’s Medium supplemented with 10% FBS. Cell lines were obtained from ATCC (Rockville, MD, USA) or from ECACC (Porton Down, Salisbury, UK). All experiments with L-lactate were performed using its sodium salt (Sigma). For short term experiments testing response to L-lactate, cells were cultured in presence of RPMI medium without glucose (Life Technologies Corporation). Conditioned medium (CM) was recovered from cells cultured in medium without FBS for at least 24 h. Evaluation of conditioned medium from WI-38 cells on PCa cell proliferation in low glucose medium was performed using the following scheme: CTR = 90% medium with 0.56 g/L glucose + 10% PC3 CM; WI38 CM = 70% medium with 0.43 g/L glucose + 30% WI-38 CM; WI38C CM = 70% medium with 0.43 g/L glucose + 30% CM from WI-38 conditioned with 30% PC3 CM. HIF-1α inhibitor, 3-(2-(4-Adamantan-1-yl-phenoxy)-acetylamino)-4-hydroxybenzoic acid methyl ester, was purchased from Calbiochem (Merck KGaA, Darmstadt, Germany).

**Figure 1:**
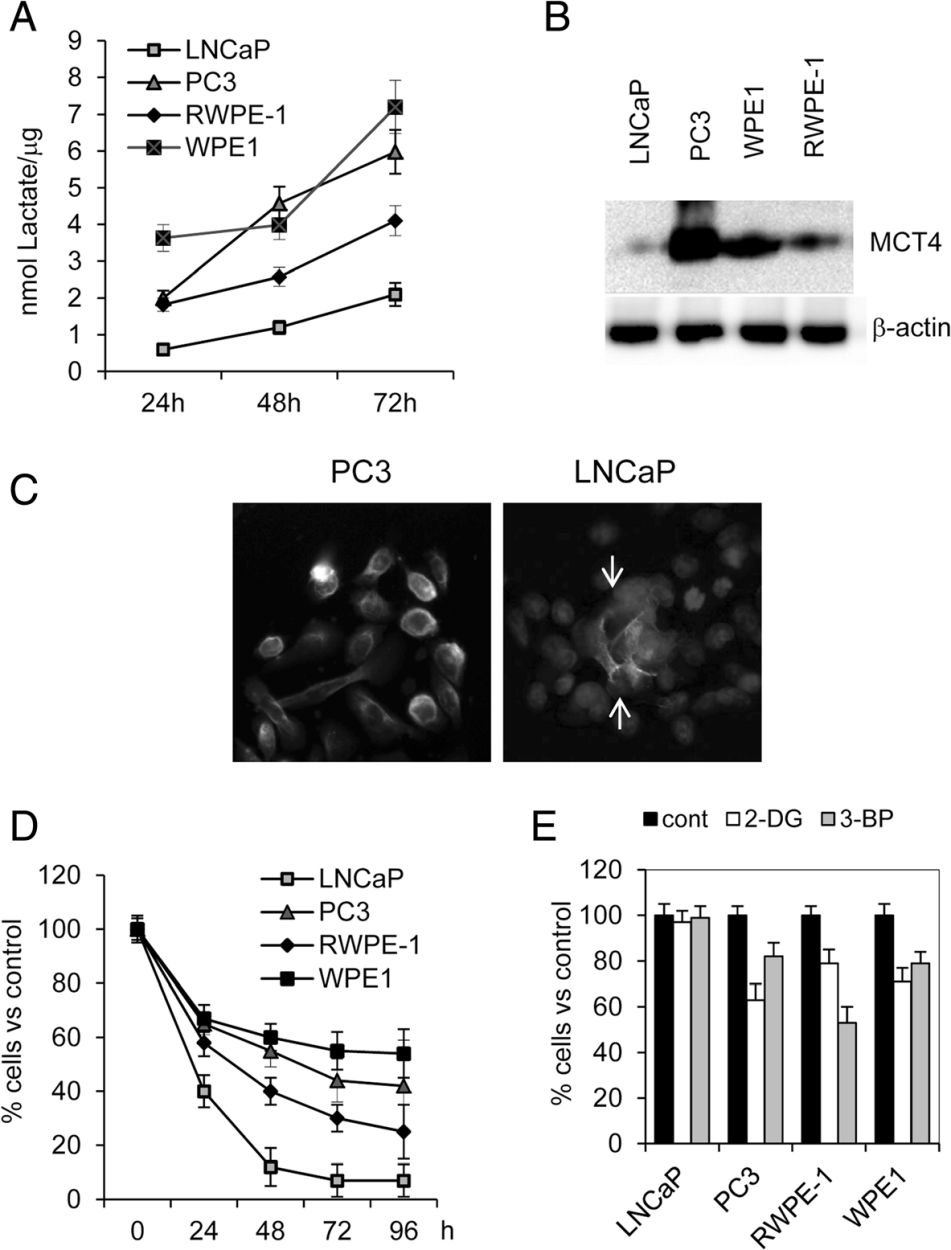
**L-lactate export and glycolytic metabolism in PCa cell lines, LNCaP and PC3, and in transformed epithelial cell lines, RWPE-1 and WPE1-NB26 (WPE1). A)** L-lactate export rate was calculated considering measurements at 24, 48 and 72 h in standard culture conditions and normalizing for total protein content (μg). **B)** Western blot analysis of MCT4 in whole cell lysates from PCa cell lines, LNCaP and PC3, and from transformed epithelial cell lines, RWPE-1 and WPE1. Beta-actin detection was utilized as loading control. **C)** Immunofluorescence detection of MCT4 in PC3 and LNCaP cells. In the image showing MCT4 expression in LNCaP cells, nuclei have been visualized by DAPI staining. Arrows indicate the positive staining for MCT4 mainly localized in the center of cell aggregate. **D)** Percentages of viable cells treated with 10 μM oligomycin for different times in comparison with untreated cells. **E)** Anti-proliferative effect of glycolysis inhibitors 2-deoxy-D-glucose (2-DG, 10 mM) and 3-bromo-pyruvate (3-BP, 50 μM). Percentages of viable cells respect to untreated cells (100%) are reported. Data represent mean values ± SD from at least three independent experiments.

### Cell proliferation assay

Cells were plated at density of 10^4^ cells/cm^2^ incubated in 5% CO_2_ at 37°C and recovered after different times of incubation. The cells were fixed for 10 min in 100% ice-cold methanol and then allowed to air-dry. The cells were stained with 0.1% w/v crystal violet in water for 10 min and washed with PBS until the excess of dye was eliminated. The stained cells were then incubated with 1% w/v SDS, 50% v/v methanol solution, and 200 μl of dissolved dye was read at 590 nm in an ELISA reader. Optical density at 590 nm is proportional to the number of attached cells, and was used to estimate the percentage of proliferation respect to control. In parallel, in order to evaluate the presence of dead cells, cell growth was also measured by direct cell counting assay, using a Neubauer hemocytometer chamber and according to trypan blue dye exclusion test. For low density growth test, cells were plated at 10 cells/cm^2^, and after 2 weeks of culture, adherent cells were stained with 0.1% w/v crystal violet. The stained colonies were photomicrographed and analyzed by number and size with the public domain software ImageJ (Rasband, W.S., ImageJ, U. S. National Institutes of Health, Bethesda, Maryland, USA, http://imagej.nih.gov/ij/, 1997–2012).

### Western blotting

Total cell lysates were obtained by incubating cells in a lysis buffer containing 1% v/v Triton, 0.1% w/v SDS, 2 mM CaCl_2_, and 100 μg/ml phenylmethyl-sulfonyl-fluoride. Protein content was determined using the Protein Assay Kit 2 (Bio-Rad Laboratory, Hercules, CA, USA). Sixty micrograms of proteins were electrophoresed in 10% SDS–polyacrylamide gel and then electrotransferred to nitrocellulose membrane (Whatmann, Dassel, Germany). The membrane was incubated with 1 μg/ml primary antibody and then with appropriate horseradish peroxidase-conjugated secondary antibodies. Protein bands were visualized using a chemiluminescent detection system (Thermo Scientific, Rockford, IL, USA) and signals were digitally acquired by Chemidoc XRS system (BIORAD). Antibodies anti-β-actin, MCT1, MCT4, COX1, β-tubulin were from Santa Cruz Biotechnology, anti HIF-1α were from Becton Dickinson (Franklin Lakes, NJ, USA), anti-AMPKalpha and p-AMPKalpha (Thr172) were from Cell Signaling Technology, Inc. (Danvers, MA, USA), anti-vimentin were from Thermo scientific (Waltham, MA, USA), anti-αSMA were from Sigma. Densitometric analysis of protein bands was performed using the ImageJ software. Relative values were calculated by comparison with experimental control, defined as 1, and normalized by the corresponding values of loading control (actin or β-tubulin).

### Quantitative RT-PCR

Total RNA was extracted from cultured cells using Genelute Mammalian Total RNA kit (Sigma) according to the manufacturer’s protocol. RNA was quantified by spectrophotometric analysis and 1 μg of RNA was used to synthesize cDNA (SuperScript III Platinum Kit, Life Technologies). Real-time PCR analysis was performed using Stratagene MX3000P personal Q-PCR in the presence of SYBR Green. The PCR reagents were provided in SuperScript III Platinum Kit (Life Technologies), and the conditions were chosen according to manufacturer’s protocol. Primers were as follows: GAPDH forward primer: 5′-GGCCTCCAAGGAGTAAGACC-3′, reverse primer: 5′-AGGGGTCTACATGGCAACTG-3′; MCT1 forward primer: 5′-TTCGGGTGGCTCAGCTCCGT-3′, reverse primer: 5′-CCTCCTCCTTGGGCCCTCCA-3′; COX1 forward primer: 5′-TCCGCTACCATAATCATCGCT-3′, reverse primer 5′-CCGTGGAGTGTGGCGAGT-3′. Mean threshold cycle (Ct) values were determined by Stratagene software using three distinct amplification curves for each gene. Relative expression of the target gene was estimated using the formula: relative expression = 2×∆Ct, where ∆Ct = Ct (target gene) – Ct (GAPDH).

### Immunofluorescence

Cells grown on coverslips (2 × 10^4^ cells/cm^2^) were fixed in 4% v/v formaldehyde in PBS for 10 min at RT and permeabilized in PBS containing 0.1% v/v Triton X-100 for 5 min at RT. Cells were then incubated with 10 μg/ml primary antibody, diluted in PBS containing 3% w/v bovine serum albumin (BSA) for 1 h at RT. After three washes with PBS, cells were treated with fluorescein-labeled IgG secondary antibody (1:100 in PBS containing 3% w/v BSA) for 30 min at RT. After extensive washings, cells were mounted with ProLong Gold antifade mounting medium (Life Technologies Corporation) and observed by fluorescence microscope equipped with digital camera (AXIOPHOT, Carl Zeiss, Oberkochen, Germany).

### Treatment with siRNA

In 10 cm culture dishes 1×10^6^ cells were plated in 8 ml antibiotic-free standard growth medium supplemented with FBS. When cells were ∼ 60% confluent, they were transfected for 5 h at 37°C with siRNA-MCT1 duplex or with scramble sequence siRNA as control of gene silencing (final concentration,100 nmol/L). Silencing experiments were performed using four distinct 22–24 nt oligo sequences from Riboxx life sciences (GmbH, Radebeul, Germany) or a pool of three target-specific 19–25 nt siRNAs. (Santa Cruz). Transfection was performed using the siRNA transfection reagent (Santa Cruz) or INTERFERin kit (Polyplus transfection, New York, NY, USA) according to the suggested protocol. Cells were cultured with siRNAs for 24 h before being subjected to specific treatments. Data in figures are the mean of the results obtained using different oligo sequences.

### Lactate assay

Conditioned media (CM) were collected, centrifuged for eliminating cells and were analysed through the L-lactate Assay Kit II according to manufacturer’s instructions (BioVision Reasearch Products, Mountain View, CA, USA). L-lactate concentration is determined by an enzyme assay, based on the L-lactate oxidation by L-lactate dehydrogenase, and the subsequent interaction with a probe which results in the formation of a coloured compound. Briefly, 0.4 μl of CM were added to each well of a 96-well plate containing 200 μl of the reaction mix and were incubated for 30 min at RT, then optical density was measured at 450 nm by Elisa reader within 1 h. The concentration was calculated by applying the sample reading to a standard curve. The L-lactate production rate was calculated normalizing the concentration with total protein content for each sample (μg) and for time (min).

### Statistical analyses

Descriptive data are presented as mean and standard deviation (SD) or median and standard error (SE) for continuous data and percentages for categorical data. Comparisons between groups was performed by using Student’s t-test or Pearson’s chi-square test. All tests for statistical significance were two tailed. All analyses were realized by using the statistical software SPSS (Texas Instruments, Chicago, IL, USA). P < 0.05 has been considered statistically significant.

## Results

### Aerobic glycolysis and L-lactate export

Both PCa cell lines, LNCaP and PC3, and TPE cell lines, non-tumorigenic RWPE-1 and tumorigenic WPE1-NB26 (WPE1), when cultured in standard culture conditions, enriched their culture medium with L-lactate but at different rates (Figure 1A). The lowest rate in L-lactate export was seen in LNCaP cells and was associated with the lowest expression of the main membrane exporter for L-lactate, monocarboxylate transporter 4 (MCT4) (Figure 1B). In addition, in LNCaP cells, but not in other prostate cell lines, MCT4 expression resulted expressed mainly in the central zone of cell aggregates (Figure 1C). This evidence, as suggested by previous studies [19], could be the result of the local hypoxia and, in consequence, of the adaptive capacity by tumor cells. TPE and PC3 cells demonstrated a more marked glycolytic phenotype respect to LNCaP cells, with PC3 cells expressing the highest amount of MCT4 (Figure 1A and B). The dissimilar dependence on aerobic glycolysis was confirmed by the dissimilar response to oligomycin, an inhibitor of oxidative phosphorylation. After 72 h of incubation with oligomycin all cell lines showed an evident reduction in cell number respect to untreated cells. LNCaP cells were almost completely killed by oligomycin after 96 h of treatment, while in the other cell lines residual viable cells were still present (Figure 1D). The utilization of aerobic glycolysis in cell lines was confirmed by the antiproliferative effect induced by specific glycolysis inhibitors 2-deoxy-D-glucose and 3-bromo-pyruvate (Figure 1E).

### Metabolic switch toward L-lactate import

**Figure 2:**
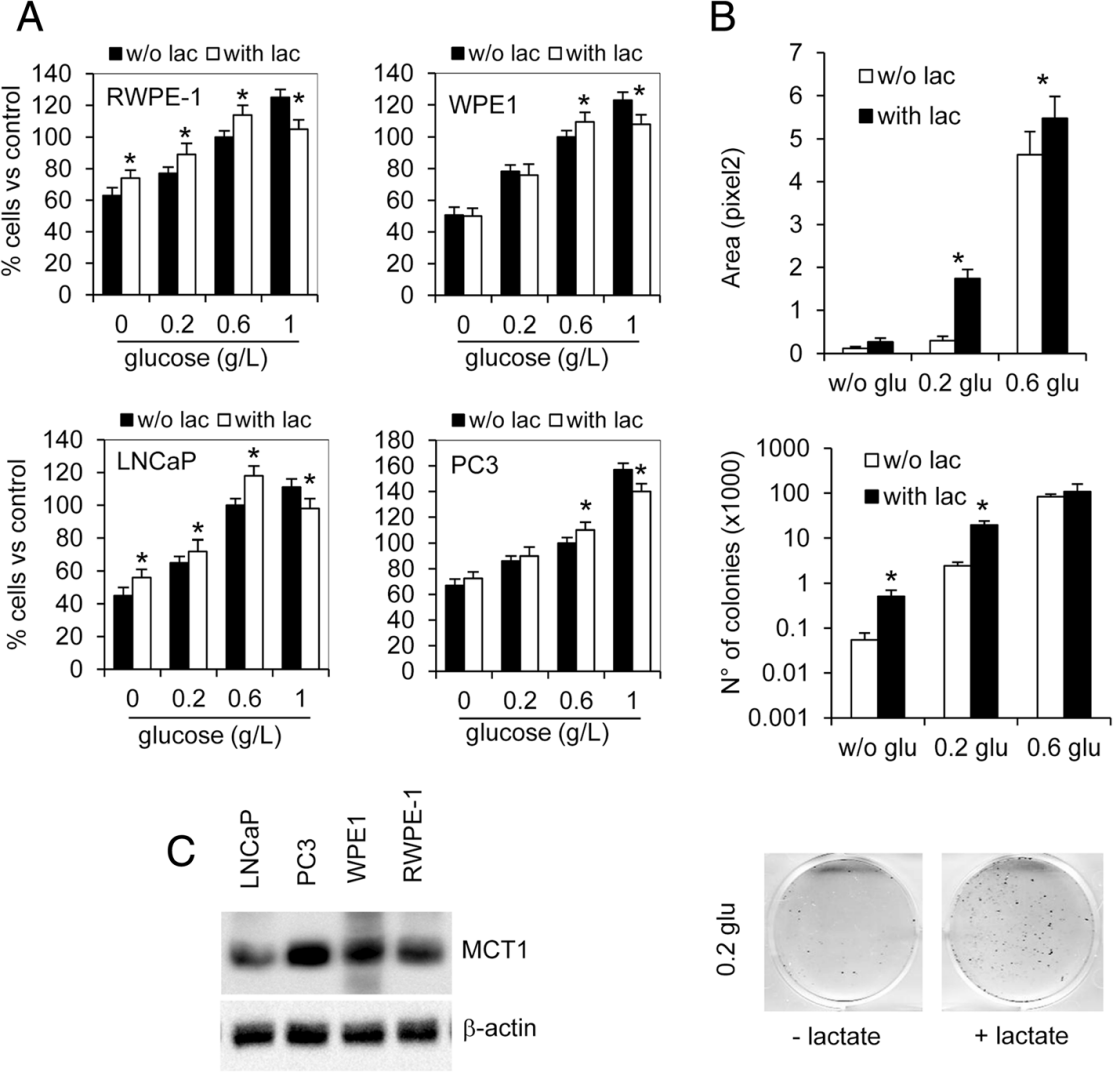
**Mitogenic and survival effect of L-lactate. A)** Proliferation assay in prostate cell lines cultured for 48 h in decreasing glucose concentrations (1, 0.6, 0.2, 0 g/L) and in presence or not of 5 mM L-lactate (lac). Cells cultured in 0.6 g/L glucose without L-lactate were chosen as reference (100%). **B)** LNCaP cells were cultured at low density for 2 weeks in decreasing glucose concentrations (0.6, 0.2, 0 g/L) in presence or not of 5 mM L-lactate (lac). At the endpoint the number of colonies and the total area covered by cells were evaluated. Representative images from LNCaP cells cultured in 0.2 g/L glucose are reported. Data represent mean values ± SD from at least three independent experiments. **C)** Western blot analysis of MCT1 in whole cell lysate from PCa cell lines, LNCaP and PC3, and from transformed epithelial cell lines, RWPE-1 and WPE1. Beta-actin detection was utilized as loading control. *P < 0.01 according to Student’s t test.

**Figure 3:**
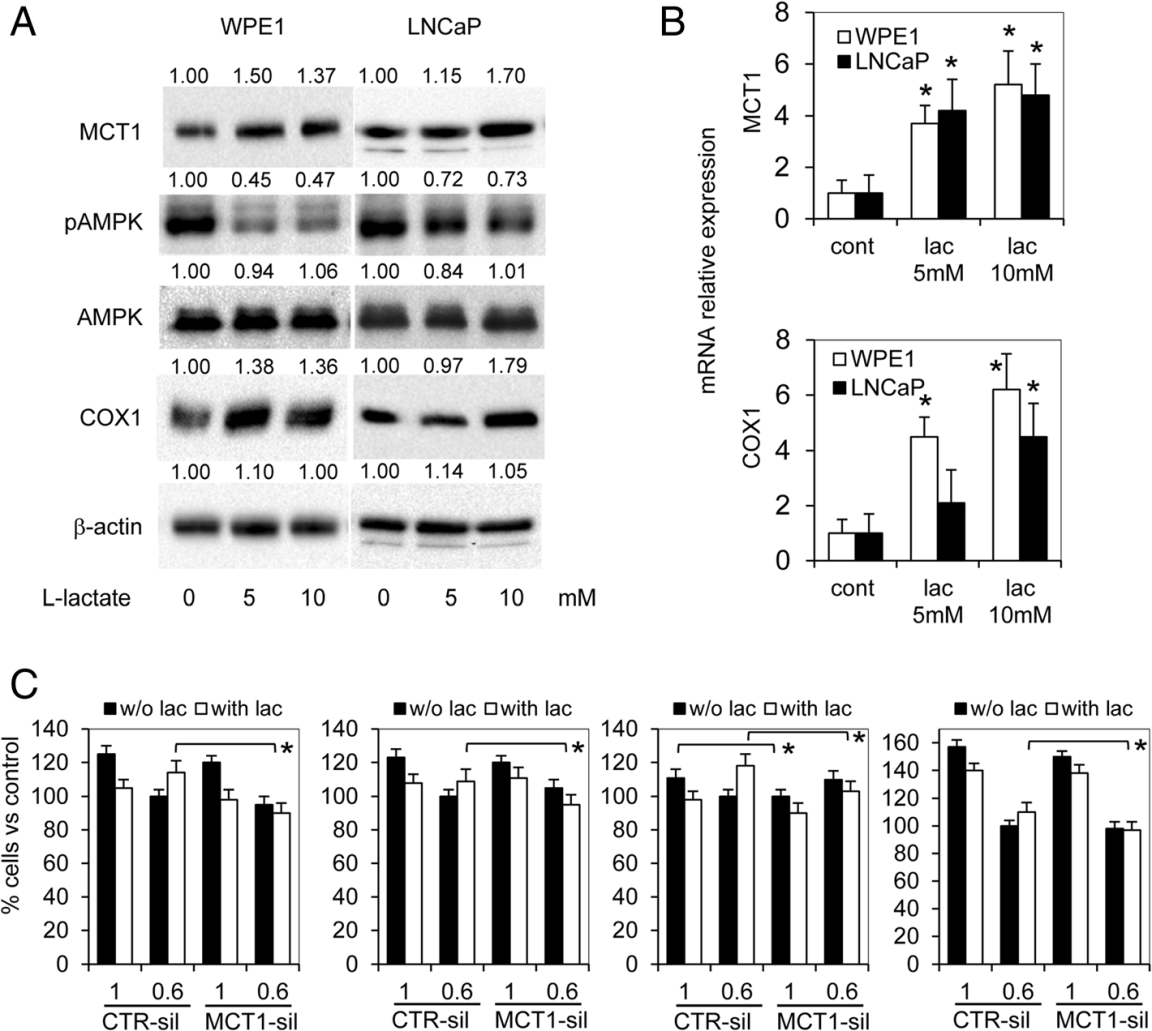
**Effects of exogenous L-lactate on prostate cells. A)** WPE1 and LNCaP cells were treated with 5 and 10 mM L-lactate for 24 h and total cell lysates were subjected to western blot analysis for MCT1, AMP activated protein kinase- alpha (AMPK), total and active form (Thr172), cytochrome c oxidase subunit 1 (COX1). Beta-actin detection was utilized as loading control. Values from densitometric analysis are shown on top of each protein band and were calculated as described in the Methods section. **B)** In the same experimental condition MCT1 and COX1 expression was detected also by quantitative RT-PCR (right histogram). **C)** Proliferation assay in control-silenced (CTR-sil) prostate cells, MCT1-silenced (MCT1-sil) prostate cells cultured for 48 h in different glucose concentrations (1, 0.6 g/L) and in presence or not of 5 mM L-lactate (lac). Cells cultured in 0.6 g/L glucose without L-lactate were chosen as reference (100%). Data represent mean values ± SD from at least three independent experiments. *P < 0.01 according to Student’s t test.

The addition of L-lactate had inhibitory effect on cell proliferation when prostate cells were cultured in normal culture conditions. On the contrary, in low glucose medium, the addition of 5 mM L-lactate sustained cell growth respect to untreated cells (Figure 2A). The mitogenic effect of L-lactate was particularly evident when cells were cultured at low density. In fact, long term treatment of LNCaP cells with 5 mM L-lactate determined a significant increase in the number and the extent of cell colonies (Figure 2B). Both TPE and PCa cells expressed the main importer for L-lactate, MCT1 (Figure 2C). In addition, in LNCaP and WPE1 cells MCT1 protein expression was stimulated by L-lactate in a dose-depend manner, and it corresponded to an increase of cytochrome c oxidase subunit 1 expression, COX1, and in the reduction of the active form of AMP-activated protein kinase alpha (AMPK) (Figure 3A). In presence of L-lactate we also observed a significant upregulation in MCT1 and COX1 mRNA (Figure 3B).

### Fibroblasts are potential sources of L-lactate in tumor microenvironment

**Figure 4:**
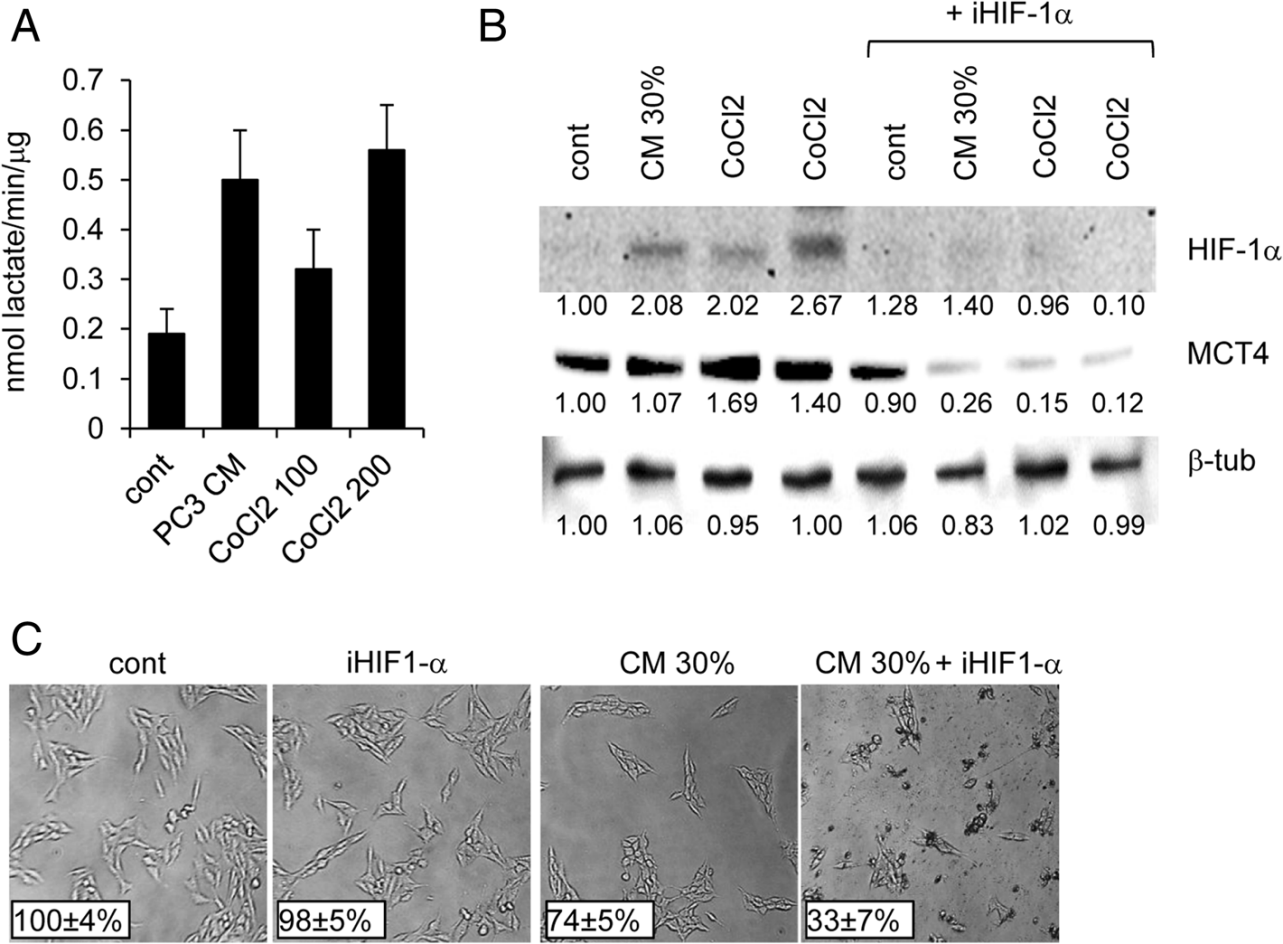
**Stimulation of glycolysis in human fibroblasts.** Diploid human fibroblasts WI-38 were cultured in presence of 30% PC3-conditioned medium (CM), or two concentrations of Cobalt(II)Chloride (CoCl2, 100 and 200 μM). **A)** After 24 h, cell media were collected and the rate of secreted L-lactate was calculated normalizing for protein content and time of culture. **B)** The same experimental conditions were repeated in presence of 50 μM HIF-1α inhibitor (iHIF-1α) and total cell lysates from fibroblasts were analyzed by western blot for the expression of MCT4 and HIF-1α. Values from densitometric analysis are shown at the bottom of each protein band and were calculated as described in the Methods section. **C)** Representative images taken by phase contrast microscopy of WI-38 cells cultured for 48 h in presence of 30% PC3 conditioned medium and 50 μM HIF-1α inhibitor. In insets the mean percentage ± SD of viable WI-38 cells from three different experiments is shown.

**Figure 5:**
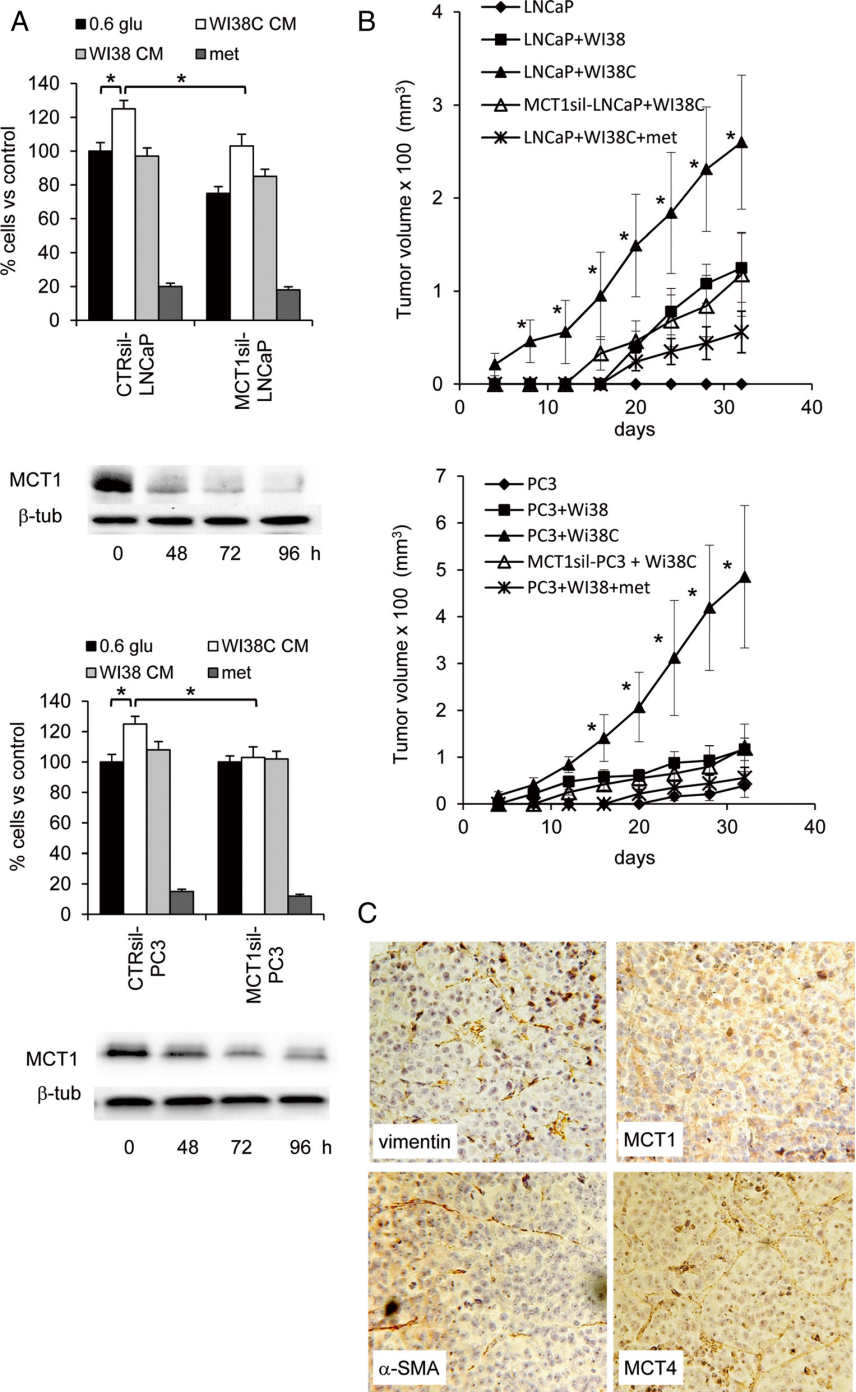
**Effect of MCT1 silencing in vitro and in vivo. A)** WI-38 conditioned medium recovered from fibroblasts treated with PC3-CM (WI38C CM) were added to PC3 and LNCaP cells cultured for 48 h in medium containing 0.6 g/L glucose. Proliferation assay was performed measuring the number of viable cells in control-silenced (CTR-sil) PCa cells, MCT1-silenced (MCT1-sil) PCa cells and using both WI38C CM and control WI-38 conditioned medium (WI38 CM). PCa cells cultured with WI38 CM were also treated with 5mM metformin. In blots time-course MCT1 expression in control and silenced PCa cells used in the proliferation assays was analyzed. Beta-tubulin detection was utilized as loading control. **B)** LNCaP and PC3 cells were injected alone or together with WI-38 cells s.c. in the flank of immunodeficient mice. Tumor growth was monitored daily and tumor growth was measured by a caliper. Experimental groups included: PCa cells alone; PCa cells injected with WI-38 fibroblasts; PCa cells injected with PC3-CM conditioned WI-38 fibroblasts (WI38C); MCT1-silenced PCa cells injected with WI38C; PCa cells injected with WI38C and treated with 50mg/kg metformin. *P>0.01 according to Student’s t test between PC3+WI38C and PC3+WI38 series. **C)** representative images of IHC analysis of xenograft tissue from mice inoculated with PC3 and WI38C (magnification 100x).

When human fibroblasts WI-38 were cultured in presence of PC3 conditioned medium (PC3-CM) they significantly increased their L-lactate export respect to fibroblasts cultured in serum-free medium (Figure 4A). In presence of 30% PC3-CM, fibroblasts upregulated MCT4 protein in a manner similar to the effect induced by the chemical inducer of hypoxia Cobalt(II) Chloride (CoCl2) (Figure 4B). Importantly, PC3 CM was able to upregulate the hypoxia-inducible factor 1-alpha (HIF-1α) respect to untreated cells. The addition of the specific HIF-1α inhibitor, 3-(2-(4-Adamantan-1-yl-phenoxy)-acetylamino)-4-hydroxybenzoic acid methyl ester, counteracted the stimulatory effect of PC3 CM and CoCl2 on MCT4 expression (Figure 4B). Interestingly HIF-1α inhibitor determined, after 48 h of incubation in presence of PC3 CM, a significant reduction of WI-38 cells respect to control cells cultured without PC3 CM, suggesting that conditioned fibroblasts developed a novel addiction for glycolysis (Figure 4C). The L-lactate enriched medium from conditioned WI-38 cells, but not medium from parental fibroblasts, was able to significantly sustain the growth of both LNCaP and PC3 cells in presence of low glucose (Figure 5A). The mitogenic effect was suppressed when PCa cells were silenced for the expression of MCT1. Significantly, MCT1 silencing was able to inhibit LNCaP, but not PC3, cell proliferation also in absence of exogenous L-lactate. The effect of L-lactate was also suppressed by metformin, a well-known AMPK agonist. Then we sought to verify if the co-inoculation with conditioned fibroblasts determined also in vivo an effective proliferative stimulus for PCa cells. In particular LNCaP cells are only moderately tumorigenic in nude mice and when inoculated sc in intact nude mice, formed tumor starting from 50 days after inoculation in about 30% of mice injected with 1*10^6^ cells. The co-injection of PCa cells with conditioned fibroblasts (WI38C) in immunodeficient mice determined an impressive acceleration in tumor growth (Figure 5B). Within the first week after the PCa cells injection the experimental group containing conditioned fibroblasts was the only group developing palpable tumors. The presence of parental fibroblasts was also able to accelerate tumor growth respect to the control group receiving only PCa cells. At endpoint (35 days) no tumors were detected in the control group injected with LNCaP cells alone. When MCT1 was silenced in PCa cells, the stimulatory effect on tumor growth was almost completely abolished (Figure 5B). A similar inhibitory effect was also exerted by metformin. When we analyzed tumor tissues from PC3 xenografts obtained by co-inoculation with WI38C, we observed that MCT1 was expressed by tumor cells while MCT4 was mainly localized in vimentin/α-SMA-positive stromal cells (Figure 5C).

### MCT1 and MCT4 expression in prostate tissue

**Figure 6:**
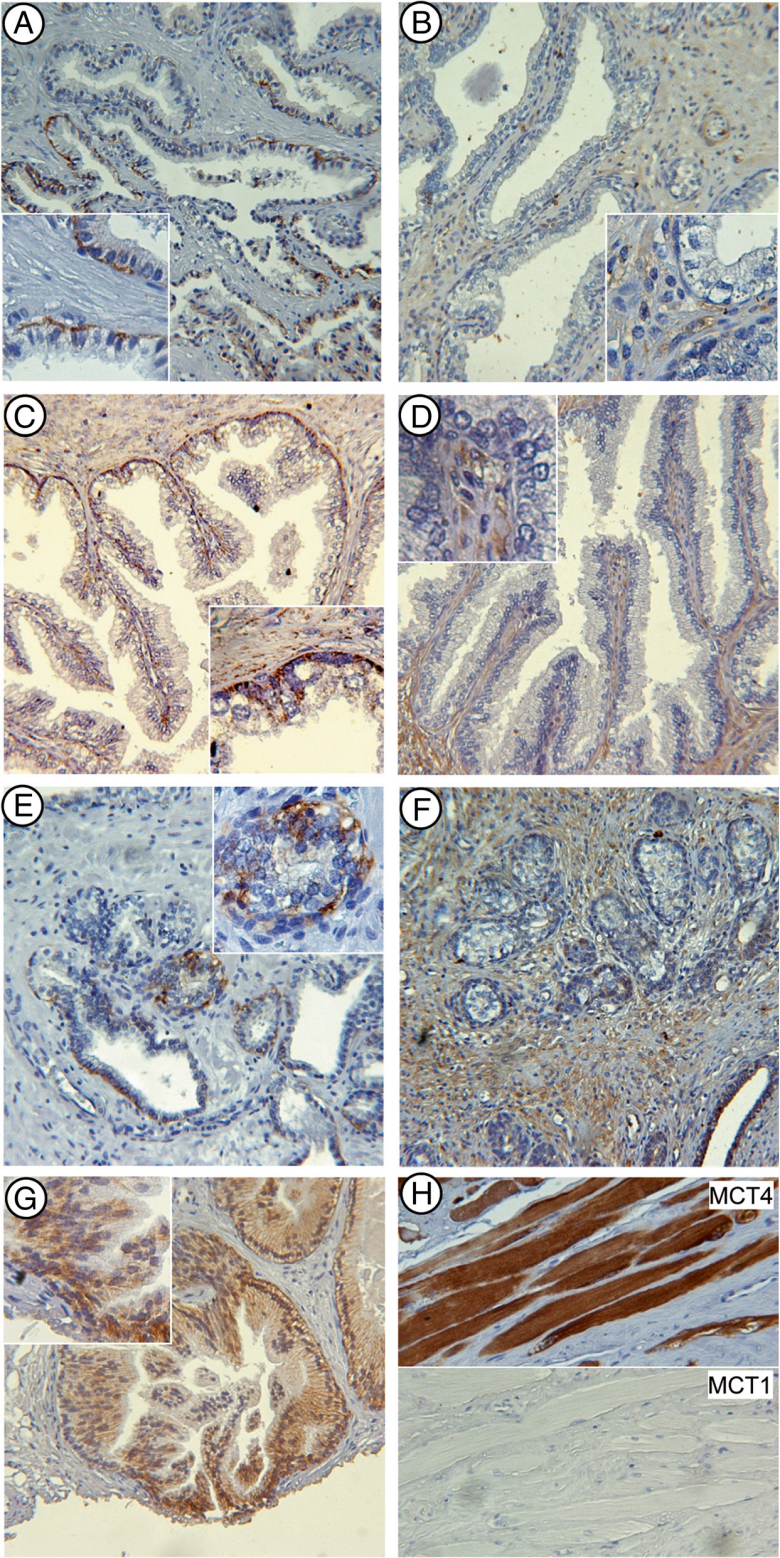
**Representative images of immunohistochemical detection for MCT1 and MCT4 in prostate tissue.** MCT1: **A)** non-neoplastic epithelium; **C)** benign hyperplasia; **E)** tumor; **G)** intraepithelial neoplastic lesion. MCT4: **B)** non-neoplastic epithelium; **D)** benign hyperplasia, **F)** tumor. **H)** MCT1 and MCT4 expression in striate muscle in the periphery of prostate gland. Images taken at 100x magnification. Inset shows a 3x magnification of the image.

In order to further support our hypothesis, we investigated the expression of MCTs in PCa (N = 80) and benign hypertrophy (BPH, N = 60) tissues. We considered age and BMI in order to avoid significant differences among patients for these parameters. PSA and testosterone, but not insulin, resulted significantly higher in PCa subjects (Table 1).Prostate specimens from radical prostatectomy (for PCa subjects) or transurethral resection of prostate (for BPH subjects) were processed for immunohistochemical detection of MCT1 and MCT4 (Figure 6). In non tumoral tissue, MCT1 was restricted to epithelial cells, mainly in basal cells and in the basolateral plasma membrane of luminal cells (Figure 6A). On the contrary MCT4 was not detectable in normal epithelial cells, but its staining was present as scattered distribution in stromal compartment (Figure 6B). A similar distribution of MCTs was also observed in BPH tissues, but frequently with a more intense staining pattern respect to non-neoplastic tissue (Figure 6C and D). In tumor tissue MCT1 expression remained restricted to carcinoma cells but its expression pattern was more diffuse involving the whole plasma membrane (Figure 6E). The increase in MCT1 expression and its re-localization on plasma membrane was particularly evident in intraepithelial lesions (Figure 6G). In tumor tissue MCT4 staining was particularly intense in tumoral stroma (Figure 6F). Interestingly we observed a strong positivity for MCT4, but not for MCT1, in striate muscle localized in peripheral prostatic gland (Figure 6H).

**Table 1:** Selected anthropometric and metabolic variables in patients with prostate cancer (PCa), and benign prostatic hyperplasia (BPH) patients

**Variable**	**PCa (N = 80)**	**BPH (N = 60)**	**P**
Age, y (mean ± SD)	64.0 ± 7.2	65.2 ± 6.5	ns
BMI, kg/m2 (mean ± SD)	27.55 ± 4.36	27.04 ± 4.59	ns
PSA, ng/mL (median ± SE)	7.12 ± 2.16	3.88 ± 0.64	<0.05
Testosterone, ng/mL (median ± SE)	5.83 ± 2.52	3.92 ± 2.12	<0.05
Insulin, mcUI/mL (mean ± SD)	8.8 ± 6.3	7.8 ± 7.6	ns
Gleason 6 (N)	45	-	
Gleason 7 (N)	35	-	

ns = not statistically significant.

We assigned a score to MCT1 and MCT4 expression according to Remmele scoring system (see Methods) and scores were categorized in low expression (< 6 points) and high expression (≥ 6 points). MCT4 expression, in both epithelial and stromal compartments, was significantly upregulated in cancer respect to hypertrophic tissue (Table 2). MCT1 expression failed to reach a statistically significant difference between PCa and BPH, however when correlation coefficients were calculated, we individuated a positive correlation between MCT4 espressed in the stroma and MCT1 expressed in tumor cells (Table 3).

**Table 2:** Pathological conditions associated with MCT1 and MCT4 expression

**Tissue**	**Expression/total number (%)**			***P-value#***
	**Null**	Low*	High**	
		**MCT1**		
BPH	48% (29/60)	47% (28/60)	5% (3/60)	0.07
PCa	31% (25/80)	56% (45/80)	13% (10/80)	
		**MCT4**		
BPH	92% (55/60)	5% (3/60)	3% (2/60)	<0.01
PCa	35% (28/80)	45% (36/80)	20% (16/80)	
		**Stromal MCT4**		
BPH	50% (30/60)	45% (27/60)	2% (3/60)	<0.01
PCa	25% (20780)	61% (49/80)	14% (11/80)	

*Low (grade <6 immunostaining); ** high (grade ≥6 immunostaining); # χ^2^ test.

**Table 3:** Pearson’s correlation coefficients calculated for epithelial MCT1, MCT4 and stromal MCT4 expression in cancer tissue

	**MCT1**	**MCT4**	**Stromal MCT4**
**MCT1**	1	−0.118 (0.715)*	0.645 (0.024)
**MCT4**	−0.118 (0.715)	1	−0.304 (0.337)
**Stromal MCT4**	0.645 (0.024)	−0.304 (0.337)	1

*P-value.

## Discussion

The processes underlying the metabolic adaptation in normal and pathologic conditions are increasingly studied. The individuation of the metabolic hallmarks that determine a proliferative advantage in energy restrictive environments will represent an important advancement in the future treatment of aggressive cancers. MCTs may play a pivotal role in metabolic adaptation because they can regulate both energetic supply and intracellular pH. We confirmed that MCT1 and MCT4 were frequently overexpressed in prostate tissue, and, importantly, we reported for the first time the upregulation of MCT4 in the stromal compartment. In order to understand the clinical impact of these differences we investigated their functional role in vitro and in vivo.

We demonstrated that the principal importer of monocarboxylate acids, MCT1 is present in non tumoral prostate epithelium, and its expression is evident in basal cells and in the baso-lateral plasma membrane of secretory cells. This evidence induced us to hypothesize that MCT1 can play an important role in feeding normal prostate epithelium. Takebe et al. described similar MCT1 expression along the basolateral membrane of crypt cells in mouse intestinal epithelium and of acinar cells in the mouse mammary glands and they suggested that intensified expression of MCT1 was associated with renewing tissues [20,21]. An elevated expression of MCT1 was usually evident in intraepithelial lesions and it was frequently associated with an increased MCT4 expression in the neighbor stromal compartment. Moreover a significant correlation between tumor MCT1 and stromal MCT4 expression exists. Also in BPH tissues, MCT4 was frequently upregulated in stromal cells while cells within hypertrophic glands showed a more diffuse pattern in MCT1 expression. This characteristic did not permit to individuate a significant association between MCT1 expression and tumor tissue respect to BPH tissue. Accordingly, other authors have individuated MCT1 expression in prostate epithelium and in PIN lesions, and they did not find any correlation with cancer progression [22,23]. A similar condition was observed also in PC3 and LNCaP xenografts where tumor masses contain a complex net of associated fibroblasts as evidenced by staining for vimentin and αSMA. In our in vivo model MCT4 was mainly expressed just by these associated fibroblasts, suggesting their early metabolic conditioning by tumor cells.

In particular, our data render plausible a mechanism based upon lactic acid shuttle between stromal and epithelial cells. In fact, in non-neoplastic prostate tissue, MCT4, the principal exporter of L-lactate, was seen only in stromal compartment and in striate muscle. It is possible that in particular pathophysiologic conditions prostate stromal cells can fuel epithelial cells. To describe this phenomenon, some authors have coined the denomination of “reverse Warburg effect” [13]. Similar energetic symbiosis is present in other organs, such as brain and ovary, also in absence of a rapid energetic expenditure, or hypoxic pressure [24,25]. It can be hypothesized that these metabolic associations have a protective role for those cells that are highly dependent on a quickly available energy supply, like in the case of neurons or oocytes.

An emerging hypothesis is that cancer-associated fibroblasts are forced to undergo aerobic glycolysis through cancer-induced mitophagy. Many are the possible causes of this phenomenon: systemic factors, such as circulating hormone, cytokines or growth factors; local factors produced by tumor cells or infiltrating inflammatory cells; local hypoxia. For example, prostate tumors have been shown to be significantly oxygen-deprived. Hypoxia has been reported to up-regulate MCT4, but not MCT1, in rat skeletal muscle [26], and in some tumor cells [27], at least in part through a transcriptional mechanism. The evidence that pO2 measurements resulted very heterogeneous in tumors with similar Gleason score is in agreement with our data indicating a variable expression of MCT4 [17]. Our results suggest that the “reverse Warburg effect” could be induced by direct interaction between cancer cells and fibroblasts. In fact, the soluble factors released by PC3 cells are sufficient to increase the release of L-lactate by human fibroblasts. The mentioned soluble factors released by PCa cells not only did induce HIF-1α, and MCT4 expression but did also stimulate in fibroblasts a HIF-1α-dependent phenotype. Indeed tumor presence may induce a stressful condition in adjacent normal cells with an increased release of catabolized nutrients, such as ketone bodies, glutamine and L-lactate [28]. As recently demonstrated this metabolic-coupling mechanism could be utilized also by prostate cancer cells promoting carcinogenesis or sustaining cancer progression, and it is based upon the release of reactive oxygen species by tumor cells [29]. Similarly, breast cancer cells can trigger aerobic glycolysis and oxidative stress in neighboring fibroblasts by secreting hydrogen peroxide [30].

Our results, in agreement with available data, support the hypothesis of a major role of MCTs in the emergence of a highly glycolytic phenotype, representing an adaptation to the hypoxic, or rapidly changing microenvironment. The up-regulation of MCT4 and the maintenance of MCT1 in the plasma membrane of PCa cells appears to be the principal adaptive mechanism to allow continuous and high glycolytic rates, by exporting the accumulating end-product, L-lactate, as well as to counteract acid-induced death [31]. PCa cells lines express both L-lactate transporters and their expression could be modulated by tumor cells according to environmental needs. This characteristic is present also in transformed prostate epithelial cells RWPE-1 and WPE1-NB26, suggesting an early energetic adaptability along tumorigenesis. However we have to consider the limitations of the available cell models. We observed that although WPE1 cells expressed higher levels of MCT4 and L-lactate export rate respect to RWPE-1 cells, these latter had a L-lactate production comparable with that of PC3 cells. Because increased glycolysis and adaptation to acidosis are key events in the transition from in situ to invasive cancer, these data are surprising. It is possible that the modality of cell transformation plays a pivotal role in determining the cellular metabolism. Accordingly, it has been demonstrated in several tumorigenic and non-tumorigenic HPV-18 infected cell lines that an appropriate level of glycolysis is an essential prerequisite for the maintenance of HPV gene expression [32].

## Conclusions

We identified MCT1 as a potential target in PCa therapy. In fact the inhibition of MCT1 transport was able to reduce the growth of PCa cells both in vitro and in vivo. However this therapeutic opportunity was evident only in specific conditions. Our data indicate that the inhibition of MCT1 is particularly dramatic for energy metabolism in presence of low glucose concentrations. Interestingly at glucose concentrations normally used in the cancer cell culture medium the addition of L-lactate resulted in the inhibition of prostate cell proliferation. It is possible that the overload of lactate in high-proliferating cells in conjuction with acidification of medium was sufficient to create sub-optimal growth conditions already after 48 h of treatment. Indeed the pro-survival and mitogenic effects of L-lactate were mainly important in low density cell culture and in sustaining the initial growth of xenografts. In these conditions, hypoxia and acidification probably play a limited role in conditioning directly tumor cell phenotype. In previous studies, targeting MCT1 has shown anticancer effects in tumor xenograft models and this phenomenon was associated with the L-lactate shuttle [14]. However other in vivo studies have failed to confirm similar results [31]. Contradictory data may be the result of the different tumor models used and of the complexity in metabolic adaptation of cancer cells. The evidence for a dual effect of L-lactate according to glucose availability needs further investigation in order to individuate the molecular sensors able to modulate intracellular energy pathways in response to environmental changes. We observed that L-lactate was able to reduce the activation of the master sensor of energy status AMPK, and this phenomenon is compatible with sustained anabolic metabolism, allowing cancer cells to escape its restraining influence on survival and growth. The inhibitory effect by metformin, observed both in vitro and in vivo, confirmed that an AMP agonist could play an important role in glucose-deprived environment compromising the metabolic adaptation by cancer cells.

Because significant differences in the expression and localization of MCTs have been detected in cancer cases with similar grading and staging, the future validation of these data could have a favorable impact on diagnosis and treatment of the more aggressive prostate cancers.

## Abbreviations

BPH: Benign prostate hypertrophy; CM: Conditioned medium; Lac: L-lactate; PCa: Prostate carcinoma; WI38C: High glycolytic fibroblasts WI-38; WPE1: Transformed epithelial cell line WPE1-NB26.

## Competing interests

Authors declare that there is not conflict of interest that has prejudiced the impartiality of the results reported or influenced the content of the manuscript.

## Authors' contributions

MB and AA designed the research; PS, CV and MC performed the research; PS and AA wrote the paper; AT and MB reviewed the paper; PC and GPG contributed to improve the scientific quality of the manuscript. All authors read and approved the final manuscript.

## Pre-publication history

The pre-publication history for this paper can be accessed here:

http://www.biomedcentral.com/1471-2407/14/154/prepub
